# Gestational trophoblastic disease and associated factors among women experiencing first trimester pregnancy loss at a regional referral hospital in central Tanzania: a cross-sectional study

**DOI:** 10.1093/inthealth/ihac015

**Published:** 2022-04-08

**Authors:** Mwajuma B Mdoe, Amos R Mwakigonja, Ipyana Mwampagatwa

**Affiliations:** Department of Clinical Nursing, School of Nursing, University of Dodoma, 259 Dodoma, Tanzania; Department of Pathology, School of Medicine, Muhimbili University of Health and Allied Sciences, 65001, Dar es Salaam, Tanzania; Department of Obstetrics and Gynaecology, School of Medicine and Dentistry, University of Dodoma, 295 Dodoma, Tanzania

**Keywords:** choriocarcinoma, gestational trophoblastic diseases, histopathology, pregnancy loss

## Abstract

**Background:**

Gestational trophoblastic diseases (GTDs) may follow any form of pregnancy or a pregnancy loss. Early detection of GTDs is important, as some benign forms of the disease may progress into a chemoresistant and metastatic disease. This study aimed at determining the frequency of GTDs among women experiencing first trimester pregnancy loss and the associated patients’ characteristics.

**Methods:**

This was a cross-sectional study that included 200 conveniently sampled women who experienced first trimester pregnancy loss from January to December 2019 at a Regional Referral Hospital in central Tanzania. The specimen obtained from products of conception were collected, formalin-fixed and paraffin-embedded and submitted for histopathological evaluation, for which haematoxylin and eosin stain was used. Data were analysed using SPSS version 23.0. The χ^2^ test was used to determine the association between categorical variables. p-Values ˂0.05 were considered statistically significant.

**Results:**

Among 200 study participants, the overall frequency of GTDs was 42 (21%). Among those with GTDs, the most common histopathological diagnosis was partial hydatidiform mole (18 [42.9%]), followed by complete hydatidiform mole (17 [40.5%]) and choriocarcinoma (7 [16.5%]). In the studied participants, only increased human chorionic gonadotropin hormone levels were found to be statistically significantly associated with GTDs (p=0.000).

**Conclusions:**

Results from this study suggest that routine histopathological evaluation of the products of conception is recommended in order to allow early detection of GTDs, including choriocarcinoma, which usually carries a poor prognosis. The histopathological reporting of choriocarcinoma among first trimester products of conception from Tanzania is novel.

## Background

First trimester pregnancy loss may be termed as abortion or miscarriage, depending on the circumstances under which it occurs. It is universally defined as a loss of pregnancy before viability (20 weeks of gestational age) or before the foetus weighs ≥500 g.^1^ Abortion complicates about 10–20% of all pregnancies worldwide, of which 10% are induced, which could be legal or illegal depending on the laws of the country, state or province in question. The literature reports that about 75% of abortions occur before the 16th week of gestational age^2^ and contribute to up to 7.9% of maternal mortality worldwide.^1^ In Tanzania, abortion and its complications account for 19% of maternal deaths,^3^ making it a problem of public health interest.

The terms abortion and miscarriage are sometimes used interchangeably to mean a pregnancy loss before viability, but abortion is usually used to refer to intentional termination of pregnancy while miscarriage is often used when there is spontaneous pregnancy loss.^2^ In either case, if there are remnant products of conception, evacuation is required. There are several methods of uterine evacuation for first trimester pregnancy loss (John, 2010).This includes medical and surgical interventions to empty the uterus and avoid complications such as continued bleeding and infection.

One of the most commonly used method of uterine evacuation is manual vacuum aspiration, which is not only easier, but also safe for evacuation of the uterus.^4,5^ In our settings, cases of abortion are commonly managed by a comprehensive post-abortion care package, which involves haemodynamic stabilization followed by dilatation and evacuation of the uterus by manual vacuum aspiration coupled with broad-spectrum antibiotics and health education on family planning use, as well as danger signs awareness and monitoring.^3^

In high-income countries, products of conception obtained from uterine evacuation are routinely submitted to the laboratory for histological evaluation with the aim of establishing the cause of the abortion and determine if further management is required should the need arise depending on histopathological findings (John, 2010). However, there is still a debate on the justification for histological diagnosis of products of conception on a routine basis.^6^ One study reported that the prevalence of gestational trophoblastic diseases (GTDs) is usually low and therefore there is no adequate scientific evidence to perform routine histological evaluations of the products of conception.^7^ However, other studies have found significant numbers of participants with molar pregnancies that need close follow-up and care to prevent future complications, including recurrent abortions as well as cancers such as choriocarcinoma.^8,9^ Some studies have revealed that the causes of abortion are likely to predict subsequent abortions and even complications, including persistent gestational trophoblastic neoplasia (GTN).^8^

In our settings, like many other resource-constrained settings, apart from the cost implications for histopathological diagnosis of the products of conception, there is no scientific justification for not performing routine histopathological diagnosis. In these settings, most abortion cases are managed empirically using a comprehensive post-abortion care package, except in a few highly suspicious cases where abnormal clinical findings may dictate histological evaluation of the products of conception. This practice has contributed to the scarcity of data on underlying pathological causes of early pregnancy losses within the country, as well as the prevalence of GTDs, as the accuracy of ultrasound in diagnosing hydatidiform moles (HMs) during the first trimester is relatively limited.^7^

The GTDs are proliferative abnormalities of trophoblasts associated with pregnancy. Histologically, it includes the pre-malignant partial hydatidiform mole (PHM), complete hydatidiform mole (CHM) and malignant invasive moles that are choriocarcinoma, placental site trophoblastic tumour (PSTT) and epithelioid trophoblastic tumour (ETT). Choriocarcinoma, PSTT and ETT are malignant invasive moles that can arise after any type of pregnancy and all are collectively known as GTNs.^10^ Recently the GTD spectrum has been expanded to also include atypical placental site nodule (APSN), as 10–15% may coexist with or develop into PSTT/ET.^11,10^ The initial stage of these diseases present like miscarriages and are often treated as common miscarriages, leading to late diagnosis with a poor prognosis.^2,12,13^ Risk factors for the development of GTDs include young age (teenage), age >35 years and previous molar pregnancy, prior miscarriage, maternal blood group A or AB, family history of GTDs and the use of birth control pills.^10,14^

Thus this study aimed to determine the frequency of GTDs and the associated factors among women experiencing pregnancy loss during their first trimester at a referral hospital in central Tanzania from January to December 2019 by conducting a systematic histopathological evaluation of first trimester products of conception, which previously was only reported from a tertiary hospital in northern Tanzania.^15^

## Methods

### Study design and setting

This was a cross-sectional descriptive study conducted at the Dodoma Regional Referral Hospital (DRRH) in the Dodoma, Tanzania. It lies in the eastern central rift valley with approximate population of 2 million people. Dodoma has the ninth highest maternal mortality rate in the country (512/100 000 live births).^16^ The hospital provides basic and comprehensive emergency obstetric care services, including comprehensive post-abortion care services. This includes evacuation of the products of conception, treatment with broad-spectrum antibiotics, family planning counselling and general health education, among many others.

### Patients’ characteristics

This study included all women who experienced first trimester pregnancy loss and agreed to sign the informed consent; and had an incomplete abortion at ≤12 weeks of their gestation age and for that reason they needed manual vacuum aspiration as part of comprehensive post-abortion care. The gestational age of study participants was obtained by participants recalling their last normal menstrual period and also by extrapolation from antenatal card.

The type of incomplete abortion was diagnosed by ultrasound and/or clinical examination. Complete abortion was diagnosed by ultrasound when an empty uterus was reported with a closed cervix, no abdominal pain and/or vaginal bleeding in a woman with a positive urinary pregnancy test; while septic abortion was diagnosed when a woman was admitted with a diagnosis of abortion and features of infection in the uterine cavity.

### Sampling method

Samples from the 200 study participants were obtained using convenient sampling methods. Consecutive collection of the products of conception was done until the required sample size was achieved during the period of 12 months.

### Collection of the products of conception

Prior to sample collection, history taking and clinical examination were performed to all participants for establishment of haemodynamic stability and diagnosis. The samples of the products of conception from the study participants were collected using manual vacuum aspiration by the gynaecologist on duty. The collected specimens were then separated from blood clots immediately and placed in a container with 10% neutral well-buffered formalin fixative and subsequently submitted for histopathology to the Central Pathology Laboratory of Muhimbili University of Health and Allied Sciences, which is incorporated in the Muhimbili National Hospital. The fixation volume was 1:10 v/v (tissue:fixative [formalin]). Before evacuation, a blood sample of the participant was collected for the purpose of analysing the β-human chorionic gonadotropin (hCG) level. The blood was drawn from venous blood into a red-top vacutainer tube. The β-hCG analysis was done at the Benjamin Mkapa Hospital Laboratory on the same day after being transported in a cool box (temperature 2–8°C). The analysis used a Chemiluminescence immunoassay (Maglumi 800 automated analyser; autodilution). The samples were centrifuged at 3000 rpm for 3 min in order to obtain serum before processing. The 500 µL of serum obtained was transferred to a sample cap and loaded into the machine for analysis. Results were analysed as normal or high, as per the American Pregnancy Association.

### Sample processing and reporting

After ensuring optimum fixation of the specimens, tissues were processed using an automated machine, then the formalin-fixed paraffin-embedded tissue blocks were stained with haematoxylin and eosin (H&E) as previously described.^17,18^ The prepared slides were then submitted for histopathological reporting as described in the quality control section below. The following criteria were used for diagnosis of a partial mole: abnormal conceptus, an embryo-foetus/foetal parts present, placenta with focal villous swelling, cistern formation, focal trophoblastic hyperplasia, unaffected villi appear normal (mixture of normal and hydropic villi) and intravillous (foetal) vasculature present. For the complete mole the diagnosis was abnormal conceptus, embryo-foetus/foetal parts absent, gross hydropic swelling of all placental villi, florid central cistern formation in the villi, pronounced trophoblastic hyperplasia, significant cytological atypia, mitotic figures and vascular villi. For choriocarcinoma, the diagnostic criteria were the presence of grossly bulky haemorrhagic tumour, complete absence of chorionic villi, trophoblastic hyperplasia, cytotrophoblast, rimmed with syncytiotrophoblasts (triphasic tumour), areas of necrosis, haemorrhage, cellular atypia and/or anaplastic trophoblastic cells.

### Data collection

A structured computerized data collection sheet was used to obtain information from the study participants, including age, marital status, level of education, occupation, parity and past obstetric history as well as clinical presentation at admission. Histopathological findings were also recorded.

### Quality control

Validity was achieved by performing calibration of the measuring instruments to control the zero error. Likewise, validation of reagents for measuring β-hCG was done by running a control. For reliability, reporting of the tissue sections for obtaining the histopathological findings was done by two experienced pathologists independently who were both blinded to the clinical information of the patients and mutual discussions were held in case of discrepancies.

### Data analysis

Data analysis was done using SPSS version 23.0 (IBM, Armonk, NY, USA). Frequency tables were used to summarize categorical variables, while continuous variables were summarized using measures of central tendency such as mean±standard deviation (SD). Association of the categorical variables was done using χ^2^ and/or Fisher's exact test as appropriate. A two-tailed p-value ˂0.05 was considered statistically significant.

### Ethical considerations, approval and consent to participate

The study was approved by the ethical research committee of the University of Dodoma (reference UDOM/DRP/REC/63, issued 7th February 2019). Permission for obtaining the samples was sought from the regional medical officer as well as the city director of Dodoma. Informed consent was obtained from every study subject for them to participate as well as allowing their products of conception specimens to be used. Patients personal information was kept strictly confidential and was not included in the questionnaires.

## Results

### Characteristics of the study participants

A total of 200 participants were recruited into the study and Table 1 shows their demographic information. The mean age of the study participants was 27±6.9 y (range 16–48). Most (73.5% [n=147]) of the participants were in the age group of 20–35 y. Most (82% [n=164]) of the study participants were married, a greater proportion (42% [n=84]) had a primary education and 45% (n=90) were self-employed. Moreover, most (68% [n=136]) of the participants had no previous history of pregnancy loss and were blood group ‘O’. Regarding the body weight of the participants, we found that 80% (n=160) were not overweight (BMI ≤25 kg/m^2^). Furthermore, most (63.5% [n=127]) of the participants had a gestational age of 10–12 weeks (mean 9±1.9) and most (65% [n=130]) of the participants had a parity of not more than 1 (mean 2±1.8).

**Table 1.** Demographic characteristics (N=200)

Variables	n	%
Age (years)		
<20	22	11.0
20–35	147	73.5
>35	31	15.5
Education level		
Informal education	15	7.5
Primary education	84	42.0
Secondary education	58	29.0
College education	43	21.5
Marital status		
Single	27	13.5
Married	164	82.0
Cohabiting	9	4.5
Occupational status		
Employed	34	17
Housewife	76	38.0
Self-employed	90	45.0

### Clinical characteristics of the study participants

Table 2 shows different clinical characteristics of the study participants. Most (58.5% [n=117]) presented with per vaginal bleeding following amenorrhea as the only presenting symptom, followed by 42 participants (21%) who had both per vaginal bleeding and lower abdominal pain (LAP) as the presenting symptoms. A total of 9 (4.5%) participants presented with only abdominal pain.

**Table 2.** Clinical characteristics of participants (N=200)

Variables	n	%
Previous pregnancy loss		
Miscarriage	36	18.0
Intrauterine foetal death	28	14.0
None	136	68.0
Maternal blood group		
A	39	19.5
B	50	25.0
O	82	41.0
AB	29	14.5
BMI (kg/m^2^)		
Normal (18.5–24.9)	160	80.0
Overweight (25.0–29.9)	40	20.0
Gestational age (weeks)		
4–6	12	6.0
7–9	61	30.6
10–12	127	63.5
Parity		
0–1	130	65.0
2–4	54	27.0
≥5	16	8.0

### Prevalence of abortions among study participants

During the entire study period we recorded 1524 abortions out of 14 519 deliveries. Thus the prevalence of abortion in our study was 10.5%. Incomplete abortions accounted for the most (64% [n=976]) of all the types of abortions in the study, with the least (2.4% [n=36]) being septic abortions.

### Histopathological results

**Figure 1.**
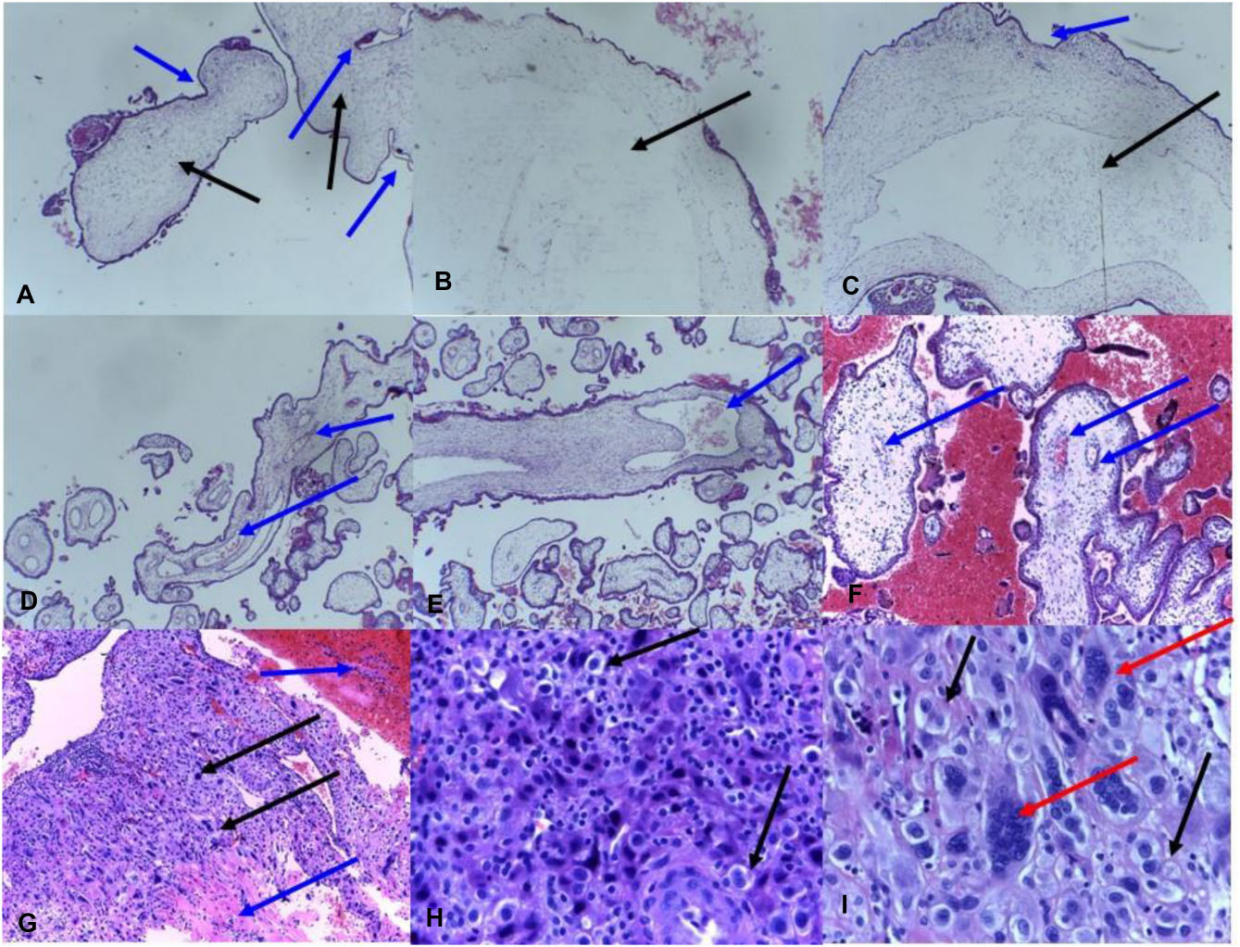
H&E microscopic sections of products of conception. **(A–I)** The histopathological results of GTDs in our study, including **(A–C)** complete moles and **(D–F)** partial hydatidiform moles as well as **(G–I)** choriocarcinoma or malignant gestational trophoblastic disease. **(A–C)** Complete hydatidiform mole. **(A)** Note the larger hydropic villi (black arrows), invaginations (blue arrows) and absence of blood vessels within the villi. Magnification ×20. **(B, C)** Note the large central cisterns within large hydropic villi. Magnification ×40. **(D–F)** Partial hydatidiform mole. **(D–F)** Note the smaller hydropic villi and the presence of foetal blood vessels within the villi (blue arrows). Magnification ×40. **(G–I)** Choriocarcinoma. **(G)** Note the increased cellularity, haemorrhage and necrosis (blue arrows) and anaplastic trophoblastic cells (black arrows). Magnification ×10. **(H)** Note the anaplastic cytotrophoblastic cells (black arrows). Magnification ×20. **(I)** Note the anaplastic syncytiotrophoblastic cells (red arrows). Magnification ×40.

Most of study participants (158 [79%]) had normal histopathological findings, as shown in Table 3. Among those with GTDs, the most common histopathological diagnosis was PHM (18 [42.9%]), followed by CHM (17 [40.5%]) and choriocarcinoma (7 [16.5%]). Table 3 and Figure 1 show the histopathological results of GTDs in our study, including CHMs, PHMs and choriocarcinoma or malignant gestational trophoblastic disease (MTD).

**Table 3.** Histopathological results for GTDs (N=200)

Histopathological patterns	n	%
Partial hydatidiform mole	18	9.0
Complete hydatidiform mole	17	8.5
Choriocarcinoma	7	3.5
None	158	79.0

**Table 4.** Association of risk factors and GTDs (N=200)

	GTD			
Variables	Present, n (%)	Not present, n (%)	χ^2^	p-Value
Age (years)			0.603	0.740
<20	6 (27.3)	16 (72.7)		
20–35	30 (20.4)	117 (79.6)		
>35	6 (27.3)	25 (80.6)		
Gestational age (weeks)			0.297	0.862
1–6	3 (16.7)	10 (83.3)		
7–9	14 (23.0)	47 (77.0)		
10–12	26 (20.5)	101 (79.5)		
HCG level (mmol/dL)				
Normal	5 (3.1)	155 (96.9)	154.076	0.000
High	37 (92.5)	3 (7.5)		
Mid-upper arm circumference (cm)			0.369	0.543
Normal (≤28)	35 (21.9)	125 (78.1)		
Overweight (>28)	7 (17.5)	33 (82.5)		
Parity			0.102	0.950
0–1	27 (20.8)	103 (79.2)		
2–4	12 (22.2)	42 (77.8)		
≥5	3 (18.8)	13 (81.2)		
Blood group			3.426	0.330
A	12 (30.8)	27 (69.2)		
B	9 (18.0)	41 (82.0)		
AB	14 (13.8)	25 (76.2)		
O	17 (20.7)	65 (79.3)		
Clinical presentation			0.472	0.091
Per vaginal bleeding	12 (30.8)	86 (73.5)		
Lower abdominal pain	0 (0.0)	9 (100.0)		
LAP and fever	4 (12.1)	29 (87.9)		
LAP and per vaginal bleeding	7 (17.1)	34 (82.9)		

### Association of patients’ characteristics and occurrence of GTDs

Table 4 presents the association of the patients’ characteristics and the occurrence of GTDs among study participants. We found that only β-hCG was strongly associated with GTDs (p=0.000). The percentage of pregnant women with GTDs who had high β-hCG was higher (92.5% versus 3.1%) than for those who had low levels. The remainder of the patients’ characteristics were not associated with the occurrence of GTDs.

## Discussion

This study reports on the prevalence of GTDs in a cohort of women who experienced pregnancy loss in their first trimester in a resource-limited facility where routine histopathological evaluation of the products of conception is not a common practice. The prevalence of HMs was 17.5%, compared with 3.5% for choriocarcinoma. These findings of a high frequency of GTDs, including a significant proportion of choriocarcinoma, among first trimester pregnancy losses in our setting highlights the importance of early screening by histopathology of products of conception, β-hCG titre measurement and follow-up during the post-abortion care period.

The prevalence of HMs reported in the our study correlates well with findings from a study at Jordan University in which the prevalence was 17%.^6^ Nevertheless, this is higher compared with studies done in India and Saudi Arabia, where the prevalence of HMs was 6% and 9%, respectively.^9,19^ The difference in the prevalence of HMs from the Indian study might be explained by the exclusion of participants, especially those who had a medical termination of a normally growing pregnancy, while in the current study it was not possible to exclude induced abortions because patients do not report them, as they are considered illegal by abortion law in Tanzania.^20^ Generally the prevalence of GTDs worldwide has been reported by some researchers to be higher.^21,22^

Furthermore, in this study, a higher frequency of HMs was seen in participants aged between 20 and 35 y of age. This result contrasts with those from a study previously done in a referral hospital in western Tanzania where HMs were found more commonly among participants between 15 and 20 y of age.^15^ These differences in the same country are difficult to account for; sample sizes for both studies were small.

Also, in this study, 97.5% of the study participants with high β-hCG levels were found to have GTDs, and this was statistically significant, which means that β-hCG might be a reliable predictor of GTDs among women with a first trimester pregnancy loss. High levels of β-hCG after evacuation of HMs may be indicative of GTN, including MTD, thus follow-up is important in order to allow for early detection and prompt initiation of chemotherapy before metastasis in case of a persistent plateau of β-hCG hormone.^23^

Again, this study shows that most (30.8%) of the participants with GTDs had blood group A. This concurs with a study done in western Tanzania in which they observed that most (20%) of the participants with GTDs were blood group A.^15^ Furthermore, another study done by Jagtap et al.^8^ in India showed a high incidence of GTDs among women with blood group A. Generally these findings are in agreement with other studies in which women with blood group A had GTDs.^8,15^ Thus this finding gives an impression that maternal blood group can explain the occurrence of GTDs. But with an understanding that the trophoblast is a product of both maternal and paternal genetic contribution, it is compelling that paternal blood group has a role to play in this disease process. However, in our study, the exploration of paternal blood groups in relation to HMs as compared with the blood groups of mothers was not done, therefore this study cannot firmly conclude whether the paternal blood group contributes to the occurrence of GTDs.

Choriocarcinoma is a malignant form of trophoblastic disease that can spread by direct invasion into the myometrium, while vascular invasion results in the spread to distant sites, most commonly the lungs, brain, liver, pelvis, vagina, kidney, intestines and spleen.^13^ Choriocarcinoma has been reported to occur in approximately 25% of cases following abortion or tubal pregnancy and 50% arise from HMs.^13^ In the current study, a high prevalence of choriocarcinoma was observed among women without a history of previous pregnancy loss whose histopathological diagnosis is unknown and would possibly be that they had a GTD in their previous pregnancies. This finding gives even more epidemiological power to the significance of histopathological diagnosis in any unexplained first trimester pregnancy loss.

A study previously done by Verma^24^ found the risk of choriocarcinoma increases with an increase in maternal age. However, the only previous Tanzanian study on the histopathology of the first trimester products of conception did not include choriocarcinoma,^15^ hence this study is the first to report on this.

Our current study shows that there is a relatively significant proportion of GTDs of 21%, including a reasonable (3.5%) proportion of choriocarcinoma cases, among women who experience pregnancy loss in their first trimester in Dodoma, Tanzania, which would have gone undetected if this study was not done. Also, GTDs were found more frequently in women <20 y of age (27.3%) and those >35 y of age (27.3%). These findings are in line with those of Hemida et al.^25^ in Egypt on the prevalence of GTDs, where they found the prevalence was higher among women <20 y of age (13.7%) and >40 y of age (28%). As expected, high levels of β-hCG in this study corresponded with the diagnosis of GTDs, which shows that patients with high β-hCG titres and abortion or miscarriage should be subjected to further workup including histopathological analysis of the products of conception. Thus our current findings highlight the role of routine histopathological evaluation of the products of conception. However, some researchers from resource-constrained countries recommend routine assessment of only those cases who are at risk of GTDs.^25^ But this does not override our opinion on the need for routine screening of all products of conception following pregnancy loss considering the high prevalence of GTDs among first trimester pregnancy losses, which will in turn improve the quality of care and outcomes. However, in this study p57 immunohistochemistry was not done to discriminate the types of GTDs, which might have somehow resulted in under or overreporting of GTDs, subsequently influencing their prevalence.

Concurrently in this study, the prevalence of abortion among study participants was done as part of a scientific facts search. The study found that the prevalence of abortion was 10.5%, which is much lower than the 21.1% reported in a previous study in Nepal.^26^ Another previous study in Nigeria, reported the incidence of abortion to be 20%, which would be in agreement with that from Nepal.^27^ The reason put forward for the high incidence of abortions among pregnant women in Nigeria included low use of family planning methods.^27^ However, the low frequency of abortions observed in our current study might be due to characteristics of the study participants, which is different from other studies as we limited our sample to only women with a first trimester pregnancy loss and restrictions by law, which make it difficult for women to disclose whether it was an induced abortion.

Furthermore, we found that the most common form of abortion in our current study was an incomplete abortion, which accounted for 64%, and septic abortion was the least, constituting only 2.4%. Obviously the low frequency of septic abortions is good news, as this is a more complicated condition which may cause mortality or lasting morbidity if mishandled. Similar findings have also been reported in a study in Egypt in which the most common type of abortion was incomplete abortion and septic abortion was least common.^6,28^ Another study done in Iraq reported similar findings, where incomplete abortion was the most common type.^29^ The reason for frequent induced abortions reported in various studies done in different settings may generally reflect the low rate of use of family planning among women of reproductive age, who normally end up with unwanted pregnancies and then opt for induced abortion, which is usually unsafe.

This is the first documentation of the histopathological evaluation of products of conception from first trimester as well as other abortions in Tanzania and the association with clinical and sociodemographic factors. As stated earlier, there is compelling scientific evidence for routine histopathological evaluation of the products of conception for women experiencing early pregnancy losses.

## Data Availability

The data set for this study contains unpublished information that can be accessed upon special request to the corresponding author.
